# Supplementary material for: The effect of antibiotic prescription in non-critically ill hospitalized patients with COVID-19: A Japanese inpatient database study
Source: PLoS One. 2025 Mar 25;20(3):e0318803. doi: 10.1371/journal.pone.0318803 (PMC11936251; doi:10.1371/journal.pone.0318803)
Supplement: Attachment — Submitted filename: Reply to Editorial comments on 2024.1.9.docx [file pone.0318803.s002.docx]

***We've checked your submission and before we can proceed, we need you to address the following issues:***

1. ***Please note that funding information should not appear in any section or other areas of your manuscript. We will only publish funding information present in the Funding Statement section of the online submission form. Please remove any funding-related text from the manuscript.***

Thank you for pointing this out. We have removed the funding information from the manuscript and included it in the cover letter, as indicated in your comment.

2. ***Please remove your figures/ from within your manuscript file, leaving only the individual TIFF/EPS image files. These will be automatically included in the reviewer’s PDF***

Thank you for your suggestion, we have removed the Figures from the manuscript.

***3. We note that you have indicated that there are restrictions to data sharing for this study. For studies involving human research participant data or other sensitive data, we encourage authors to share de-identified or anonymized data. However, when data cannot be publicly shared for ethical reasons, we allow authors to make their data sets available upon request. For information on unacceptable data access restrictions, please see http://journals.plos.org/plosone/s/data-availability#loc-unacceptable-data-access-restrictions.***

***Before we proceed with your manuscript, please address the following prompts:***

***a) If there are ethical or legal restrictions on sharing a de-identified data set, please explain them in detail (e.g., data contain potentially identifying or sensitive patient information, data are owned by a third-party organization, etc.) and who has imposed them (e.g., a Research Ethics Committee or Institutional Review Board, etc.). Please also provide contact information for a data access committee, ethics committee, or other institutional body to which data requests may be sent.***

***b) If there are no restrictions, please upload the minimal anonymized data set necessary to replicate your study findings to a stable, public repository and provide us with the relevant URLs, DOIs, or accession numbers. Please see http://www.bmj.com/content/340/bmj.c181.long for guidelines on how to de-identify and prepare clinical data for publication. For a list of recommended repositories, please see https://journals.plos.org/plosone/s/recommended-repositories. You also have the option of uploading the data as Supporting Information files, but we would recommend depositing data directly to a data repository if possible.***

***Please update your Data Availability statement in the submission form accordingly."***

Thank you for your suggestion. a) is relevant. The data is held by a third-party organization, MDV, and the contact person regarding this data is Yuki Santo. Their email address is [santo_yuki@mdv.co.jp]. If necessary, please contact the provided contact information. The data availability statement in the submission form has been updated. Please confirm.
